# Counterfactual Thinking in Tourette's Syndrome: A Study Using Three Measures

**DOI:** 10.1155/2014/256089

**Published:** 2014-11-30

**Authors:** Stefano Zago, Adriana Delli Ponti, Silvia Mastroianni, Federica Solca, Emanuele Tomasini, Barbara Poletti, Silvia Inglese, Giuseppe Sartori, Mauro Porta

**Affiliations:** ^1^U.O.C. Neurologia, IRCCS, Fondazione Ospedale Maggiore Policlinico, Milan, Italy; ^2^Tourette Centre and Department of Functional Neurosurgery, IRCCS Galeazzi, Milan, Italy; ^3^Department of Neurology and Laboratory of Neuroscience, “Dino Ferrari Center”, University of Milan Medical School, IRCCS Istituto Auxologico Italiano, Milan, Italy; ^4^Dipartimento di Psicologia Generale, Università degli Studi di Padova, Italy

## Abstract

Pathophysiological evidence suggests an involvement of frontostriatal circuits in Tourette syndrome (TS) and cognitive abnormalities have been detected in tasks sensitive to cognitive deficits associated with prefrontal damage (verbal fluency, planning, attention shifting, working memory, cognitive flexibility, and social reasoning). A disorder in counterfactual thinking (CFT), a behavioural executive process linked to the prefrontal cortex functioning, has not been investigated in TS. CFT refers to the generation of a mental simulation of alternatives to past factual events, actions, and outcomes. It is a pervasive cognitive feature in everyday life and it is closely related to decision-making, planning, problem-solving, and experience-driven learning—cognitive processes that involve wide neuronal networks in which prefrontal lobes play a fundamental role. Clinical observations in patients with focal prefrontal lobe damage or with neurological and psychiatric diseases related to frontal lobe dysfunction (e.g., Parkinson's disease, Huntington's disease, and schizophrenia) show counterfactual thinking impairments. In this work, we evaluate the performance of CFT in a group of patients with Tourette's syndrome compared with a group of healthy participants. Overall results showed no statistical differences in counterfactual thinking between TS patients and controls in the three counterfactual measures proposed. The possible explanations of this unexpected result are discussed below.

## 1. Introduction

Tourette's syndrome (TS) is a neuropsychiatric disorder characterized by chronic multiple motor tics and one or more phonic/vocal tics, defined as semivoluntary, repetitive, and stereotyped movements and vocalization [1]. In the* Diagnostic and Statistical Manual, *now in its fifth edition (DSM-5), TS is defined as a tic disorder characterized by an early onset before the age of 18 years and is not secondary to the administration of drugs known to cause motor side effects or to the presence of other disorders [2]. Investigation using neuroimaging and neurophysiologic techniques suggests that pathophysiology of tic is associated with changes in brain function and structure within the cortico-striato-thalamo-cortical pathway [3–5].

There is still debate as to the extent to which TS is associated with cognitive impairment. In general, subtle cognitive changes have been detected in tasks involving verbal fluency, planning, attention shifting, working memory, cognitive flexibility, and social reasoning [6–9]. Some authors argue that uncomplicated TS is associated with mild deficits in tasks involving inhibitory processes [10]. An important strand of current research relates to social cognition, as it is becoming evident that some aspects of social reasoning involved in decision-making are altered in TS [9]. These studies report that patients with TS exhibited significantly poorer performance than controls in tasks involving “theory of mind,” the ability to reason about mental states, for example, beliefs and emotions. Eddy et al. [9] suggested that TS patients show subtle differences in social cognition which can be tapped by suitably sensitive measures and could reflect dysfunctions in frontostriatal pathways involving the ventromedial prefrontal cortex. Instead, Eddy and Cavanna [11] argued that a number of TS patients may actually be more sensitive than controls to the same emotional and social cues. They also stated that TS patients may have “*a greater awareness of potential behaviors linked to negative affective consequences *…* which could prompt the hypothesis that an increase in CFT may be seen in TS.*” However, there is no empirical evidence that CFT deficit occurs in the course of TS.

CFT is the capacity to “do otherwise” in situations and is critically influenced by the ability to mentally represent possible behaviours and probable scenarios. This human skill is known as counterfactual reasoning, or thinking (CFT), and consists of the “*tendency for people to imagine alternative outcomes to events that have actually occurred*” [12]. Even if the concept of CFT traditionally refers to mental representations of past events, the capacity to represent alternative behaviours can also deal with the future “*… if I do this, I'm sure that … if instead I do something else, it could happen that …*” [13]. CFT plays an important role in cognitive functioning in daily living, being connected to a wide range of psychological and behavioural processes such as decision-making, planning, problem-solving, and experience-driven learning, all cognitive processes which are mainly linked to the prefrontal lobes. CFT usually takes the form of conditional statements, with an antecedent “*If only …*” and a consequent “*… then ….*” The antecedent describes an action or a decision made by someone, and the consequent shows a state of being [14]. Alternative faculties may be better than reality (upward counterfactuals) or worse (downward counterfactuals). Upward and downward CFT have different consequences for our decisions, feelings, and moods. Upward CFT, often makes people feel worse, but seems to have a preparative function. Thinking on how things could have been better generates feelings of regret and leads to finding more desirable outcomes for the future, eliciting consequent actions. To imagine how things could have been worse (downward CFT) can make people feel better about the same event and this seems to have a regulatory function, aiding in coping and having an ameliorating affect [15].

Recent neuroscientific evidence suggests that CFT is active in different brain regions. Barbey et al. [20], on the basis of fMRI studies in healthy people, suggested that counterfactual representations for reasoning about the past or predicting the future depend on “structured event complexes” that is the ability to shift from perceiving the immediate environment to an alternative, imagined perspective. These “structured event complexes” are neurally supported by the medial prefrontal cortex. van Hoeck et al. [21], in a fMRI study, demonstrated that CFT involves a brain network related to conflict detection, action monitoring, adaptive control, and physical causality. Particularly involved are the posterior medial cortex and lateral prefrontal cortex, as well as areas related to memory, such as both temporal lobes, the left temporal gyrus, and the left cerebellum. Moreover, CFT strongly recruits the inferior parietal lobule. Kulakova et al. [22] in an fMRI study compared CFT with hypothetical conditions (i.e., which activate only the suppositional model, making no statement about factual events) across visual and aural modalities. They showed activation in the right occipital cortex (cuneus) and right basal ganglia (caudate nucleus) during counterfactual sentence processing, with the occipital activation present in visual and auditory stimulus presentation.

Studies conducted with patients with frontal lobe damage provide evidence that they cannot generate a normal level of different behaviours and their choices are made using a very limited number of alternatives [23, 24]. They also show an inability to foresee the possible negative consequences of their own actions. In particular, CFT disturbances have been documented in patients with focal prefrontal lobe damage, especially of the orbitofrontal region and in some neurological and psychiatric populations related to frontostriatal circuit dysfunctions, such as Parkinson's disease [25], Huntington's disease [26], and schizophrenia, in which alterations of the frontal lobe have been observed [27, 28]. Notably, an abnormal increase in CFT, in the sense of mental rumination (i.e., repetitive thinking about a topic), has been typically seen in anxious and depressed patients [29].

Two main methods have been proposed for the quantitative evaluation of CFT [25]: firstly, a direct method consisting of the generation of CFT statements starting from negative autobiographical events. The subjects are asked to recount an unpleasant event from their lives (e.g., a particular failure at school or work) and subsequently they are asked to generate possible CFT alternatives that could have changed the course of events and canceled the negative one. Secondly, a tool for evaluating CFT indirectly is the* counterfactual inference test* [27], which assesses CFT by attributing feelings in response to different scenarios.

Here we compared patients with TS and healthy controls on measures of CFT. This represents a novel direction in TS literature since difficulty with CFT has been hypothesized in this population but has not previously been examined.

## 2. Method

### 2.1. Subjects

Forty-eight consecutive adult patients with TS were recruited from two centres, the Movement Disorders and Tourette Centre of the Department of Functional Neurosurgery, IRCCS Galeazzi, Milan, Italy, and the San Marco Hospital of Zingonia in Bergamo, Italy. The group was composed of 14 females and 34 males. The average age of patients was 33.9 (SD 11.7), with a mean age onset of tic at 8.2 years (SD 4.4), ranging from 18 to 60 years of age. Ten patients out of 48 already had an activated deep brain stimulation (DBS) implant.

Patients met the DSM-V [2] and World Health Organization criteria for TS. Tic frequency and types, as an indicator of TS severity, were assessed through the* Yale Global Tic Severity Rating Scale* (*YGTSS*) [30]. A control group of 46 age, education, and sex matched orthopaedic outpatients without anamnestic neurological/psychiatric diseases was recruited from the IRCCS Galeazzi (see Table 1). Information about comorbidities in the TS group (OCD, ADHD, LD, mood disorders, etc.) is reported in Table 2.

### 2.2. Materials

#### 2.2.1. Cognitive and Behavioural Measure

To evaluate the cognitive status of each patient, in addition to the* mini-mental state examination* (MMSE) [17] we administered two frontal lobe tests: the* Frontal Assessment Battery *[19], which includes some subtests measuring inhibition, and the* Verbal Phonemic Fluency Test* [18]. We also administered the* Dysexecutive Questionnaire, Subject Form* [16], a self-reporting measure concerning dysexecutive behaviour in everyday life (see Table 1).

#### 2.2.2. Counterfactual Thinking Measures

CFT was evaluated using three measures, proposed by Hooker et al. [27].

(1) *Spontaneous counterfactual generation test* is focused on frequency of CFT in response to a personal, real-life event. Participants were asked to recall a negative personal event; they were given three minutes to analyse this event in detail. Negative events, as opposed to positive events, were used because a previous study had shown that spontaneous CFT is more frequent in such events [31]. Participants were then asked if, recalling their personal life event, they had had any thoughts of how things might have gone differently, that is, thoughts of “if only” or “what if.” Responses were recorded and the number of counterfactual thoughts was tabulated. Counterfactual thoughts were defined as any thoughts that offered a different alternative action than which might have been taken [27].
(2) *Counterfactual inference test (CIT)* analyzes the ability to use CFT in order to make inferences. It is based on past research about those factors that have been shown to heighten CFT. Kahneman and Tversky [32], for example, found that outcomes preceded by unusual as opposed to typical actions enhance CFT; moreover, events that seem spatially or temporally “almost” to have occurred also increase CFT [33]. Thus, CIT is a forced choice test with four questions: for each question, events experienced by two individuals are presented and three response options are given. The two subjects experience similar outcomes, but the circumstances between them differ so that one should think “if only” to a greater extent than the other (see Table 3).
(3) The third CFT test focuses on the* influence of anticipated counterfactual regret on behavior*, testing the hypothesis that the anticipation of regret influences decision-making. Participants randomly received one of three versions (A, B, and C) of a scenario, which was specifically designed by Hetts et al. [34]. Subjects were asked to read it carefully and to imagine that the scenario was happening to them. In all versions, participants were asked to imagine that they had just arrived at the office the morning of an important job interview:

*Imagine that you are driving to an office where you have an important job interview for which you have waited for a long time. Further, imagine that after parking the car, you are walking to the office in a bit of rush because you do not want to be late for the interview. On the way to the office, however, you get a strange feeling that you may have left your car door unlocked. Even thinking hard about it, you cannot be absolutely certain whether or not you locked the door.*

One-third of participants received the scenario exactly as described above (*version C*), that is the neutral scenario, which does not evoke any feeling of regret. To the contrary, the remaining participants received one of the two nonneutral scenarios (*version A, version B*). One-third of participants were asked to imagine the following end to the scenario (*version A*):

*Think for a minute about how upset you would feel if you decided not to go back to check your car, and later your car was burgled.*

The last third of participants were asked to imagine an alternative end to the same scenario (*version B*):

*Think for a minute about how upset you would feel if you decided to go back to check your car and ended up being late for the interview and missing the chance to attend it.*

Different endings in versions A and B are aimed at inducing a specific CFT that evokes a feeling of regret, influencing participants' decision-making. In fact, the anticipation of counterfactual regret is assumed to influence later behavioral intentions. Prior to a decision, participants induced to consider a potential regret (versions A and B) will be more likely to choose behaviors that minimize the chances of experiencing that negative regret.

After imaging themselves in these situations, participants were asked to decide whether they would go back to check their car or go straight to the office for the job interview.

Finally, we also assessed the participants' level of* confidence*, asking them to state the accuracy of their choices on a scale from 0 (totally incorrect) to 5 (totally correct).

## 3. Results

### 3.1. Cognitive Tests

**Figure 1:**
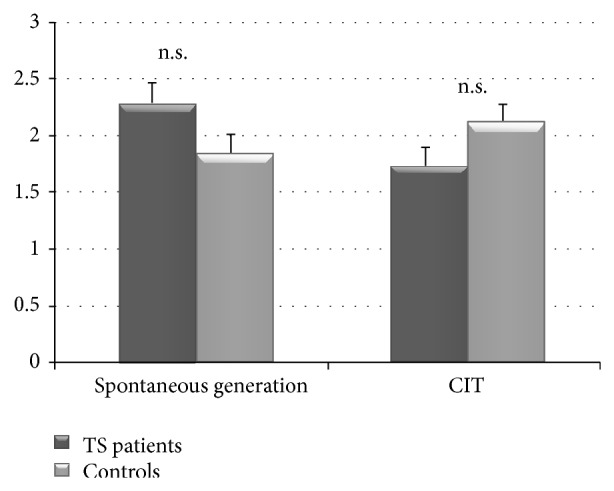
Correct responses produced by TS patients and in control subjects in* spontaneous counterfactual generation test* and* counterfactual inference test (CIT).*

**Figure 2:**
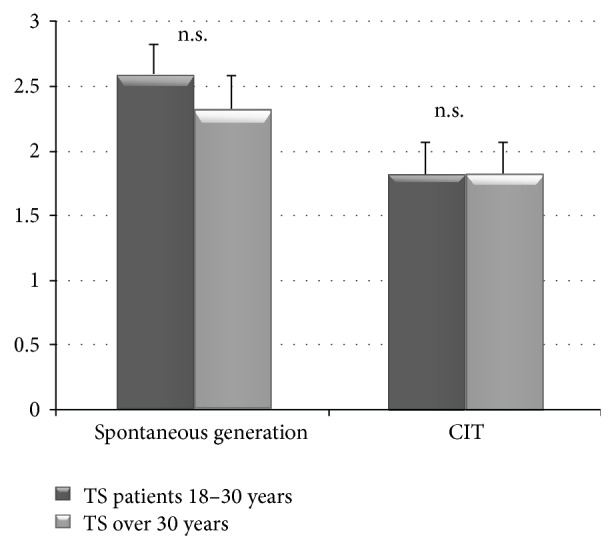
Correct responses produced by under 30-year-old and over 30-year-old TS patients in* spontaneous counterfactual generation test *and* counterfactual inference test (CIT).*

As reported in Table 1, TS performance was within the normal range on MMSE and on the two frontal measures (*Verbal Phonemic Fluency Test, Frontal Assessment Battery FAB*). The DEX-S total mean score was 24.9, indicating only a moderate dysexecutive functioning, as proposed by Pedrero-Pérez et al. [35]. There were no influences of gender and education on the cognitive performances. In addition, no differences emerged between TS patients and controls in the three CFT tasks. In particular, TS patients reported a comparable number of mental alternatives in response to recalling a negative personal event as did controls (Spontaneous Generation Mean: TS = 2.3; Controls = 1.9; *P* = 0.076 ns) (Figure 1). Moreover, participants obtain similar scores on CIT, a test analysing the ability to use CFT in order to make inference (CIT total score TS = 1.7; Controls = 2.1; *P* = 0.08 ns). The two groups differed neither on the test focused on the influence of anticipated counterfactual regret on behavior, nor on the level of confidence shown (*Regret P* = 0.64;* Confidence Level P* = 0.072 ns) (Figure 2). No differences were found in the patients with DBS implant.

To analyze if age among TS patients played a part in performance, we divided the TS sample into two subgroups one under 30 years old and the second over. Using the Mann-Whitney statistical test, we found that three measures (YGTSS, FAB, and Verbal Fluency) revealed better scores among TS patients over thirty years old (see Table 4).

## 4. Discussion

Over the last decade, there has been an accumulating body of evidence showing that CFT is sustained by a brain network in which a main role is played by the prefrontal cortex. Patients with focal prefrontal lobe damage or with neurological and psychiatric diseases related to frontal lobe dysfunction (e.g., Parkinson's, Huntington's, and schizophrenia) show CFT impairments. A deficit in CFT has only been hypothesized but never examined in TS.

Thus, the aim of this study is to analyze, for the first time, CFT in a sample of 48 adults with TS, compared to a group of healthy control participants. We administered three CFT measures: one focused on the frequency of counterfactual thinking in response to a personal real-life event, one showing that affective and judgmental reactions regarding social events are influenced by counterfactual thinking, and one on the influence of anticipated counterfactual regrets on behaviour. Data demonstrated that the TS group was able to generate as comparable a numbers of alternatives, in response to recalling a negative event, as were controls. TS patients were also as skilful as controls in using CFT in order to make inferences regarding hypothetical social events. This could be considered an unexpected result if we look at previous studies on patients with frontostriatal damage, such as Parkinson's and Huntington's, in which an impairment in CFT was detected. However, some accounts can be offered to explain this result.

When compared with PD and HD patients, TS patients display a dissimilar involvement of the basal ganglia and different evolutions of the cognitive condition over time. It is well documented that in early adulthood, roughly three-quarters of TS patients will have greatly diminished childhood tic symptoms and over one-third will be tic free [36]. In addition, compensatory changes in brain structure and function have been observed in children with TS [37].

We can speculate that also the cognitive status, including executive functions, may follow a similar trend in TS. Differently to conditions involving progressive subcortical neurodegeneration, such as PD and HD, in TS the potential heterogeneity determines varied neural abnormalities and a clinical course in which changes in cognitive function may range from mild to absent. Varied prefrontal circuitry may be involved in TS and such differences in the syndrome leave room for the possibility that in a considerable number of patients prefrontal functions are partially or totally preserved. Our TS patients could be considered to represent a group whose executive functions are relatively intact, especially when measured through CFT tasks that do not appear to evaluate these abilities in depth. Moreover, another explanatory hypothesis takes into account the ability of working memory. The production of CFT requires an important load of the working memory: to evoke a counterfactual thought, it is necessary to hold the memory of a past unpleasant event in the working memory long enough to compare what actually happened with the counterfactually derived alternative. To hold such complex information in the working memory requires resisting interference, which is a well-documented process mediated by the prefrontal lobes. Thus, CFT tests are strongly mediated by working memory abilities. Several studies showed how TS patients do not differ significantly from healthy subjects on measures of working memory [11, 38] and such data could be explained by the fact that these patients performed well on CFT tests. On the contrary, due to cognitive interference, PD or HD patients are unable to efficiently produce counterfactual models and to compare these models with memories of the adverse events. However, further studies are needed to strengthen support for this explanation. Another possible explanation concerns social cognition and the “*Theory of Mind*”; several studies have underlined how some aspects of social reasoning involved in decision-making are altered in uncomplicated TS patients and how TS patients can show alterations in these abilities, which are strongly linked to frontal lobe functioning [9, 11]. Our CFT tests, however, do not include other individuals or social and interpersonal relationships in the scenario and it is possible that our tasks do not sufficiently assess CFT in depth in TS patients. That is why a CFT test, which includes production of alternatives, not only by patients but also by other individuals, could highlight the difficulty of TS patients in generating alternatives to past events. Moreover, recent studies have shown that TS is linked to a mixed pattern of preserved and impaired performances on social cognition tests [39]. Hence, further studies are needed to better understand the contribution of social aspects to everyday functioning, especially in childhood and adolescence with TS. To our knowledge, no studies on social cognition in TS at a younger age are available; however, in future the application of social cognition tasks, already used with children with other pathologies (e.g., [40]), could be applied to younger TS patients.

## Figures and Tables

**Table 1:** Demographic, clinical, and neuropsychological data of TS patients (*n* = 48) and healthy controls (*n* = 46). Data are expressed as mean (SD).

Factors	TS patients (*N* = 48)		Controls (*N* = 46)		*P*
	M (SD)	*n*	M (SD)	*n*	
Age (years)	33.9 (11.7)	48	30.5 (9.3)	46	0.12
Gender (female/male)	14/34	48	14/32	46	0.93
Education (years)	10.9 (3,2)	48	10.9 (3.3)	46	0.90
Right/left-handed	43/5	48	40/6	46	0.74
Onset of the disease (years)	8.3 (4.4)	45			
DBS (yes/not)	10/38	48			
YGTSS (total score)	36.9 (24.7)	48			
DEX-S (total score)	24.9 (14.8)	47	cut off >18	[16]^*^	
MMSE (total score)	28.2 (1.6)	48	cut off 23.80	[17]^*^	
Verbal fluency (total score)	27.2 (8.9)	48	cut off >17	[18]^*^	
FAB (total score)	15.3 (1.4)	48	cut off >13.50	[19]^*^	

^*^95% of normal subjects scored above the cut-off.

**Table 2:** Presence of comorbidity and associated symptoms in TS patients. Data are expressed in percentage (%).

Comorbidities/coexisting symptoms	TS patients
OCB/OCD (YBOCS total score ≥16)	75%
SIB	22%
ADHD	67%
DSA	35%
Behavioural disorders	69%
Depression	30%
Anxiety	73%

**Table 3:** The *counterfactual interference test *(Hooker et al. 2003 [27]).

Scenery		Response
(1)	Janet is attacked by a mugger only 10 metres from her house. Susan is attacked by a mugger 1 kilometre from her house. Who is more upset by the mugging?	(**a**)** Janet** (b) Susan (c) Same (d) Cannot tell

(2)	Ann gets sick after eating at a restaurant she often visits. Sarah gets sick after eating at a restaurant she has never visited before. Who regrets their choice of restaurant more?	(a) Ann (**b**)** Sarah** (c) Same (d) Cannot tell

(3)	Jack misses his train by five minutes. Ed misses his train by more than one hour. Who spends more time thinking about the missed train?	(**a**) **Jack** (b) Ed (c) Same (d) Cannot tell

(4)	John gets into a car accident while driving on his usual way home. Bob gests into a car accident while trying a new way home. Who thinks more about how his accident could have been avoided?	(a) John (**b**)** Bob** (c) Same (d) Cannot tell

*Note*. Correct or normative answers to questions are in bold: (1) (a), (2) (b), (3) (a), and (4) (b).

**Table 4:** Differences between under 30 years old and over 30 years old TS patients.

Factors	TS patients 18–30 y (*N* = 22)	TS over 30 y (*N* = 26)	*P*
YGTSS (total score)	49.1 (23.4)	26.6 (21.2)	0.001^*^
MMSE (total score)	28.1 (1.5)	28.4 (1.6)	0.51
Verbal fluency (total score)	22.5 (8.8)	31.1 (7.1)	0.0005^*^
FAB (total score)	14.7 (1.2)	15.7 (1.3)	0.01^*^
DEX (total score)	28.4 (14.2)	22.1 (15.1)	
DBS (1)	18 (81.8%)	20 (76.9%)	0.74^#^
Gender (2)	15 (68.2%)	19 (73.1%)	0.76^#^
Education	10.8 (3.3)	10.9 (3.1)	0.87
CFT			
Spontaneous generation (number of alternatives)	2.6 (1.1)	2.0 (1.3)	0.12
CIT (total score)	1.8 (1.2)	1.65 (1.2)	0.64
Regret (1)	12 (54.6%)	16 (61.5%)	0.77^#^
Confidence level (0–5)	3.45 (0.96)	3.92 (1.02)	0.110

^*^Significant; ^#^Fisher exact test.
